# Current status of stroke in hemodialysis patients on a remote island

**DOI:** 10.1371/journal.pone.0288731

**Published:** 2023-09-08

**Authors:** Hikaru Nakamura, Takeshi Hiu, Yasuhito Yamamoto, Shinya Oda, Tsuyoshi Izumo, Takayuki Matsuo

**Affiliations:** 1 Department of Neurosurgery, Nagasaki University Graduate School of Biomedical Sciences, Nagasaki, Japan; 2 Department of Internal Medicine, Nagasaki Kamigoto Hospital, Nagasaki, Japan; Lithuanian University of Health Sciences, LITHUANIA

## Abstract

**Objectives:**

Hemodialysis patients have a higher incidence of stroke than healthy individuals. Hemodialysis patients living on remote islands are subject to additional distance and transportation difficulties. Therefore, we aimed to study the association between stroke and hemodialysis in patients living on remote islands.

**Materials and methods:**

We conducted a retrospective cohort study based on the medical records of maintenance hemodialysis patients in Shinkamigoto-Cho, Nagasaki, Japan, between June 1, 2005, and June 31, 2022. The clinical characteristics, probability of hemorrhagic stroke, acute ischemic stroke-free rate, and survival probability with or without a history of anticoagulant/antiplatelet use were evaluated. The survival probability among the hemorrhagic stroke, acute ischemic stroke, and non-stroke groups was also evaluated.

**Results:**

This study involved 142 patients. Nine patients (6.3%) had intracerebral hemorrhage, one (0.7%) had subarachnoid hemorrhage, eight (5.6%) had acute ischemic stroke, and 124 (87.3%) had no stroke. The number of patients with severe disabilities (modified Rankin Scale 5/6) was significantly higher in the hemorrhagic stroke group. The probability of hemorrhagic stroke and acute ischemic stroke-free rate, or survival probability with or without a history of anticoagulant/antiplatelet use, were not significantly different. The acute ischemic stroke group was not associated with a lower survival probability than the other groups. The hemorrhagic stroke group had a significantly lower survival probability than the acute ischemic stroke group.

**Conclusions:**

This is the first study to report the status of stroke in hemodialysis patients living on remote islands, thus providing valuable information for improved stroke management in such patients.

## Introduction

**Fig 1:**
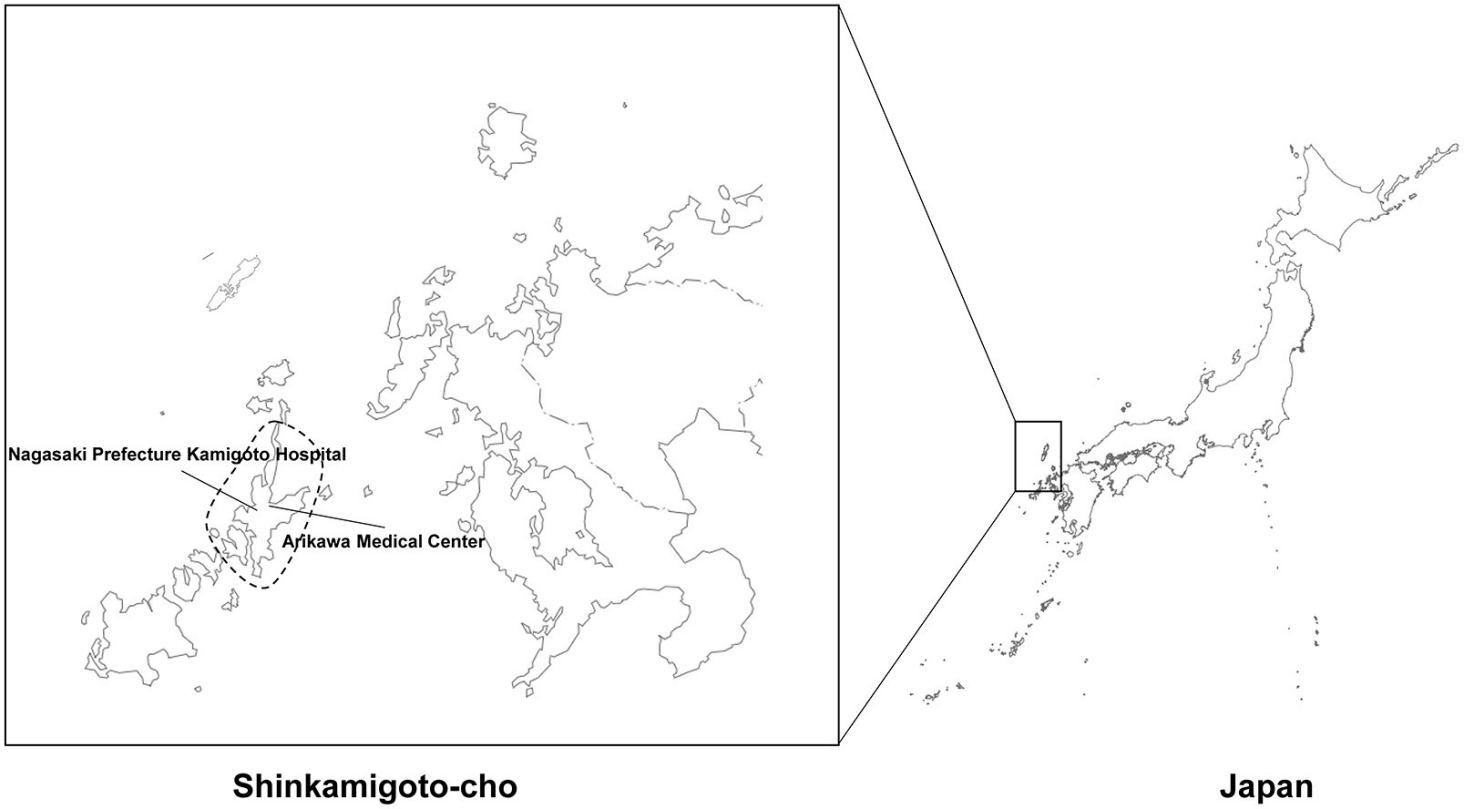
Geographical location of Nagasaki Kamigoto Hospital and Arikawa Medical Center. Modified reprint from the Geospatial Information Authority (GSI) of Japan with permission and modified from GSI under CC BY license, original copyright 2022.

Nagasaki has 51 inhabited islands, the most significant number in Japan, with three main islands: Goto, Iki, and Tsushima. Shinkamigoto-Cho is a region located on Goto (Fig 1). Shinkamigoto-Cho has an area of 213.94 square kilometers and a population of 17,503 according to the 2020 census. The aging rate of those aged ≥65 is 42.7%, which is significantly higher than the national average of 28.0% [1], making it one of the most aged populations in the world.

Patients with chronic kidney disease have an increased risk of cardiovascular events and cerebrovascular diseases as renal function declines [2–4], which can be more severe with reduced prognosis after onset [5]. In particular, the incidence of stroke in hemodialysis patients is 17.2/1,000 person-years, which is higher than that in healthy individuals [6]. Additionally, intracerebral hemorrhage (ICH) occurs in 0.6–1.0% of patients with chronic kidney disease annually, which is 5–10 times that in healthy individuals [7–11].

There are many areas where stroke specialists or facilities are absent on remote islands. It is often necessary to transport patients by helicopter to advanced medical institutions on the mainland after the onset of symptoms. Therefore, for managing stroke, many issues are unique to remote islands, such as distance and time constraints. Despite these conditions, there have been no reports on the association between stroke and hemodialysis patients with a high risk of stroke living on remote islands.

In Shinamigoto-Cho, only two facilities can perform hemodialysis, Nagasaki Kamigoto Hospital and Arikawa Medical Center, and these facilities treat all hemodialysis patients in the region. We aimed to study the association between stroke and hemodialysis in patients from this remote island using the medical records of these two facilities.

## Materials and methods

### Ethics

The study was carried out in accordance with The Code of Ethics of the World Medical Association (Declaration of Helsinki) and approved by Nagasaki Kamigoto Hospital (approval no. 04–03). The requirement for written informed consent was waived owing to the retrospective nature of the study and use of deidentified data.

### Patients

**Fig 2:**
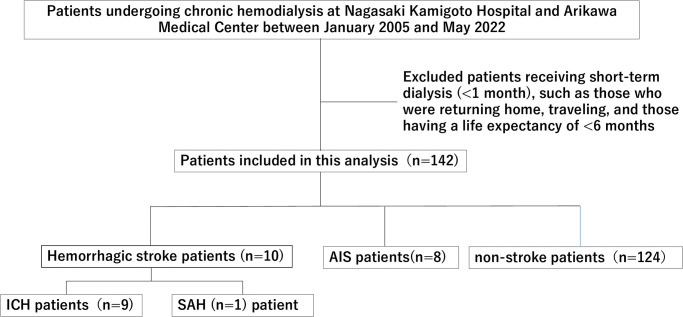
Flow diagram of patient selection. We performed a retrospective cohort study based on patients’ medical records on maintenance hemodialysis at Nagasaki Kamigoto Hospital and Arikawa Medical Center between June 1, 2005, and June 30, 2022. The inclusion criterion was end-stage renal disease requiring maintenance hemodialysis. Patients with short-term dialysis treatment (<1 month), follow-up patients returning home or traveling, and patients with short life expectancies (<6 months), such as those with terminal cancer, were excluded. AIS, acute ischemic stroke; ICH, intracerebral hemorrhage; SAH, subarachnoid hemorrhage.

We performed a retrospective cohort study based on patients’ medical records on maintenance hemodialysis in Nagasaki Kamigoto Hospital and Arikawa Medical Center between June 1, 2005, and June 30, 2022. The inclusion criteria were end-stage renal disease requiring maintenance hemodialysis. Patients with short-term dialysis treatment (<1 month), follow-up patients returning home or traveling, and patients with short life expectancies (<6 months), such as those with terminal cancer, were excluded (Fig 2). The date of the first maintenance hemodialysis was considered the initial date of entry into this cohort study.

Stroke types were considered at the initial onset and classified into hemorrhagic stroke and acute ischemic stroke (AIS). Hemorrhagic stroke was further classified into ICH and subarachnoid hemorrhage (SAH). Ischemic stroke was classified according to the Trial of Org 10172 in Acute Stroke Treatment (TOAST) classification [12] by two physicians with experience in neurosurgery. The amount of hematoma was calculated as follows [13]:

$$\begin{array}{l} \text{Estimated}\;\text{hematoma}\;\text{volume}\;\left(\text{mL}\right)=\{\text{maximum}\;\text{diameter}\;\text{of}\;\text{high-density}\;\text{area}\;\left(\text{cm}\right)\\ \times \;\text{maximum}\;\text{short}\;\text{diameter}\;\left(\text{cm}\right)\times \text{number}\;\text{of}\;\text{slices}\;\text{with}\;\text{high-density}\;\text{area}\}\times 1/2 \end{array}$$

We evaluated the clinical characteristics and collected the following information from medical records: sex, age at the start of hemodialysis, duration of hemodialysis (months), primary disease requiring hemodialysis (diabetes mellitus, glomerulonephritis, or nephrosclerosis), stroke risk factors (hypertension, diabetes mellitus, hyperlipidemia, and atrial fibrillation), and history of smoking and anticoagulant/antiplatelet use. The modified Rankin Scale (mRS) was used to evaluate stroke patients at discharge.

### Statistical analysis

Data are presented as mean ± standard deviation (SD). Between-group comparisons were performed using the Mann–Whitney *U* test and Fisher’s exact test or chi-square test. The end point was defined as stroke incidence or death. Stroke events were defined as rapidly developing signs of focal or global disturbance of cerebral function (transient ischemic attack was not included). Using the Kaplan–Meier method and the log-rank test, we examined the ICH-/AIS-free rate and the survival probability with or without anticoagulant/antiplatelet use. We also compared the survival probability among the hemorrhagic stroke, AIS, and non-stroke groups. Statistical significance was set at p<0.05. Statistical analyses were conducted using EZR, a modified version of the R commander, designed to add statistical functions frequently used in biostatistics [14].

## Results

Table 1 shows the baseline characteristics of the study population. In total, 142 patients were included in this study. Among them, nine (6.3%) patients had ICH (ICH patients), one (0.70%) had SAH (SAH patient), eight (5.6%) had AIS (AIS patients), and 124 (87.3%) did not have stroke (non-stroke patients). Smoking history was significantly higher in the hemorrhagic stroke group than in the AIS group. There were no significant differences in other incidences among the three groups.

**Table 1:** Baseline characteristics of the study population.

	Total (n = 142)	A: non-stroke (n = 124)	B: Hemorrhagic stroke (n = 10)	C: AIS (n = 8)	p value (A vs. B)	p value (A vs. C)	p value (B vs. C)
・Sex, n (%)							
Male	91 (64.1)	81 (65.3)	8 (77.8)	2 (25.0)	0.49	0.05	0.05
Female	51 (35.9)	43 (34.7)	2 (22.2)	6 (75.0)	0.49	0.05	0.05
・Age at HD initiation (years)	65.8 (SD±13.6)	66.1 (SD±13.5)	66.8 (SD±13.9)	61.1 (SD±15.4)	0.87	0.32	0.44
・Duration of HD (months)	97.5 (SD±84.4)	95.4 (SD±86.3)	82.9 (SD±52.2)	149.1 (SD±79.7)	0.65	0.09	0.05
・Primary disease of HD							
DM, n (%)	47 (30.1)	40 (32.3)	5 (55.6)	2 (25)	0.30	1.0	0.37
Glomerulonephritis, n (%)	31 (21.8)	26 (21.0)	2 (22.2)	3 (37.5)	1	0.37	0.61
Nephrosclerosis, n (%)	25 (17.6)	24 (19.4)	0 (0)	1 (12.5)	0.21	1.0	0.44
Other, n (%)	14 (9.9)	13 (10.5)	1 (0)	0 (0)	1.0	1.0	1.0
Unknown, n (%)	25 (17.6)	21 (16.9)	2 (22.2)	2 (25.0)	0.68	0.63	1.0
・Comorbidity							
HT, n (%)	122 (85.9)	105 (84.7)	9 (88.9)	8 (100)	1.0	0.60	1.0
DM, n (%)	58 (40.8)	51 (41.1)	5 (55.6)	2 (25.0)	0.74	0.47	0.37
HL, n (%)	62 (43.7)	56 (45.2)	4 (44.4)	2 (25.0)	1.0	0.47	0.64
AF, n (%)	35 (24.6)	30 (24.2)	3 (33.3)	2 (25.0)	0.71	1.0	1.0
・Smoking history	53 (37.3)	48 (38.7)	5 (44.4)	0 (0)	0.52	0.05	0.04
・Anticoagulant (warfarin) use history, n (%)	25 (17.6)	22 (17.7)	2 (22.2)	1 (12.5)	1.0	1.0	1.0
・Antiplatelet use history, n (%)	52 (36.6)	47 (37.9)	4 (44.4)	1 (12.5)	1.0	0.26	0.31
・Type of antiplatelet							
Aspirin	42 (29.6)	37 (29.8)	4 (40)	1 (12.5)	0.50	0.44	0.31
Clopidogrel	13 (9.2)	13 (10.5)	0 (0)	0 (0)	0.60	1.0	N/A
Cilostazol	7 (4.9)	6 (4.8)	0 (0)	1 (12.5)	1.0	0.36	0.44
DAPT	11 (7.7)	10 (8.1)	0 (0)	1 (12.5)	1.0	0.51	0.44
・Both anticoagulant and antiplatelet agents	10 (7.0)	8 (6.5)	2 (20)	0 (0)	0.16	1.0	0.48

ICH, intracerebral hemorrhage; SAH, subarachnoid hemorrhage; AIS, acute ischemic stroke; p value (A vs. B), refers to a 2-way comparison (non-stroke, hemorrhagic stroke); p value (A vs. C), refers to a 2-way comparison (non-stroke, AIS); p value (B vs. C) refers to a 2-way comparison (hemorrhagic stroke, AIS); HD, hemodialysis; SD, standard deviation; DM, diabetes mellitus; HT, hypertension; HL, hyperlipidemia; AF, atrial fibrillation; N/A, not applicable; DAPT, dual antiplatelet therapy.

The site of ICH was putaminal in five (50.0%) patients, lobar in two (20.0%) patients, and intraventricular and posterior fossa in one (10.0%) patient each. The remaining patient (10.0%) had SAH. Among them, the mean putaminal hematoma volume was 73.6 mL (SD ±78.9), the mean lobar hematoma volume was 52.5 mL (SD ±47.7), and the posterior fossa hematoma volume was 38.8 mL. No patients were transported to advanced medical institutions or required surgical intervention. Only two (20.0%) patients were discharged (mean duration of discharge: 31.5 [SD ±34.1] days) (Table 2).

**Table 2:** Characteristics of hemorrhagic stroke patients.

Site of hematoma	n (%)	Size of hematoma (ml)
・Putaminal	5 (50.0)	73.6 (SD±78.9)
・Lobar	2 (20.0)	52.5 (SD±47.7)
・Intraventricular	1 (10.0)	
・Posterior fossa	1 (10.0)	38.8 (SD±0)
・SAH	1 (10.0)	
Transportation to advanced medical institutions	0 (0)	
Surgical intervention	0 (0)	
Discharge destination to home	2 (20)	
Days to discharge	31.5 (SD±34.1)	
Total	10	

SD, standard deviation; SAH, subarachnoid hemorrhage; HT, hypertension.

According to the TOAST classification, atherothrombotic brain infarction was observed in four (50.0%) patients, cardiogenic cerebral embolism in two (25.0%) patients, lacunar infarction in one (12.5%) patient, and another type of ischemic stroke in one (12.5%) patient. No patients were transported to advanced medical institutions, were administered recombinant tissue-type plasminogen activator (rt-PA), or required endovascular treatment. Three (37.5%) patients were discharged (mean duration of discharge: 34.5 [SD ±18.9] days) (Table 3).

**Table 3:** Characteristics of AIS patients.

Stroke subtype	n (%)
・ATBI	4 (50.0)
・CCE	2 (25.0)
・LI	1 (12.5)
・Other	1 (12.5)
Transportation to advanced medical institutions	0 (0)
rt-PA intervention	0 (0)
EVT intervention	0 (0)
Discharge destination to home, n (%)	3 (37.5)
Days to discharge	34.5 (SD±18.9)
Total	8

AIS, acute ischemic stroke; ATBI, atherothrombotic brain infarction; CCE, cardiogenic cerebral embolism; LI, lacunar infarction; rt-PA, recombinant tissue-type plasminogen activator; EVT, endovascular treatment; SD, standard deviation.

**Fig 3:**
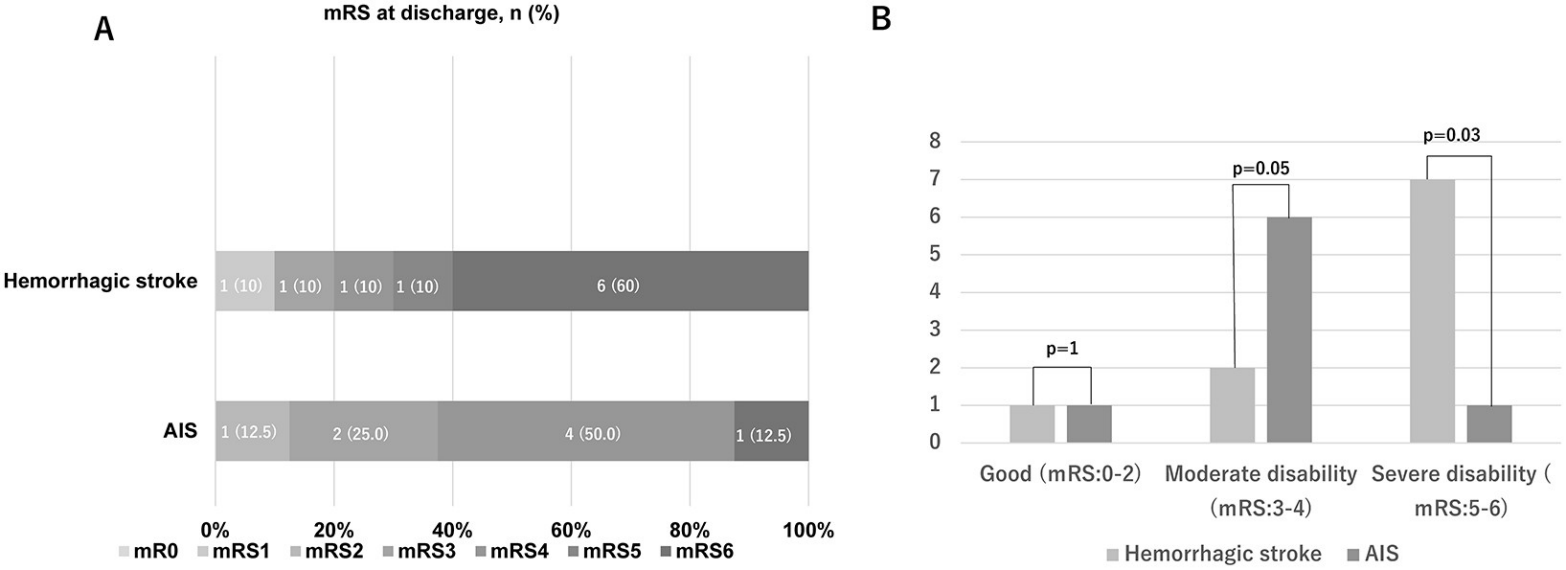
(A) Distribution of mRS scores at discharge. (B) Comparison of mRS scores at discharge between hemorrhagic stroke and AIS patients. Six (60.0%) patients in the hemorrhagic stroke group had mRS 6, only one (12.5%) patient with AIS had mRS 6 at discharge. The proportion of patients with severe disability (mRS 5/6) at discharge was significantly higher in the hemorrhagic stroke group (p = 0.03). AIS, acute ischemic stroke; mRS, modified Rankin Scale.

Six (60.0%) patients in the hemorrhagic stroke group had mRS 6, while only one (12.5%) patient with AIS had mRS 6 at discharge (Fig 3A). The proportion of patients with severe disability (mRS 5/6) at discharge was significantly higher in the hemorrhagic stroke group (p = 0.03) (Fig 3B).

**Fig 4:**
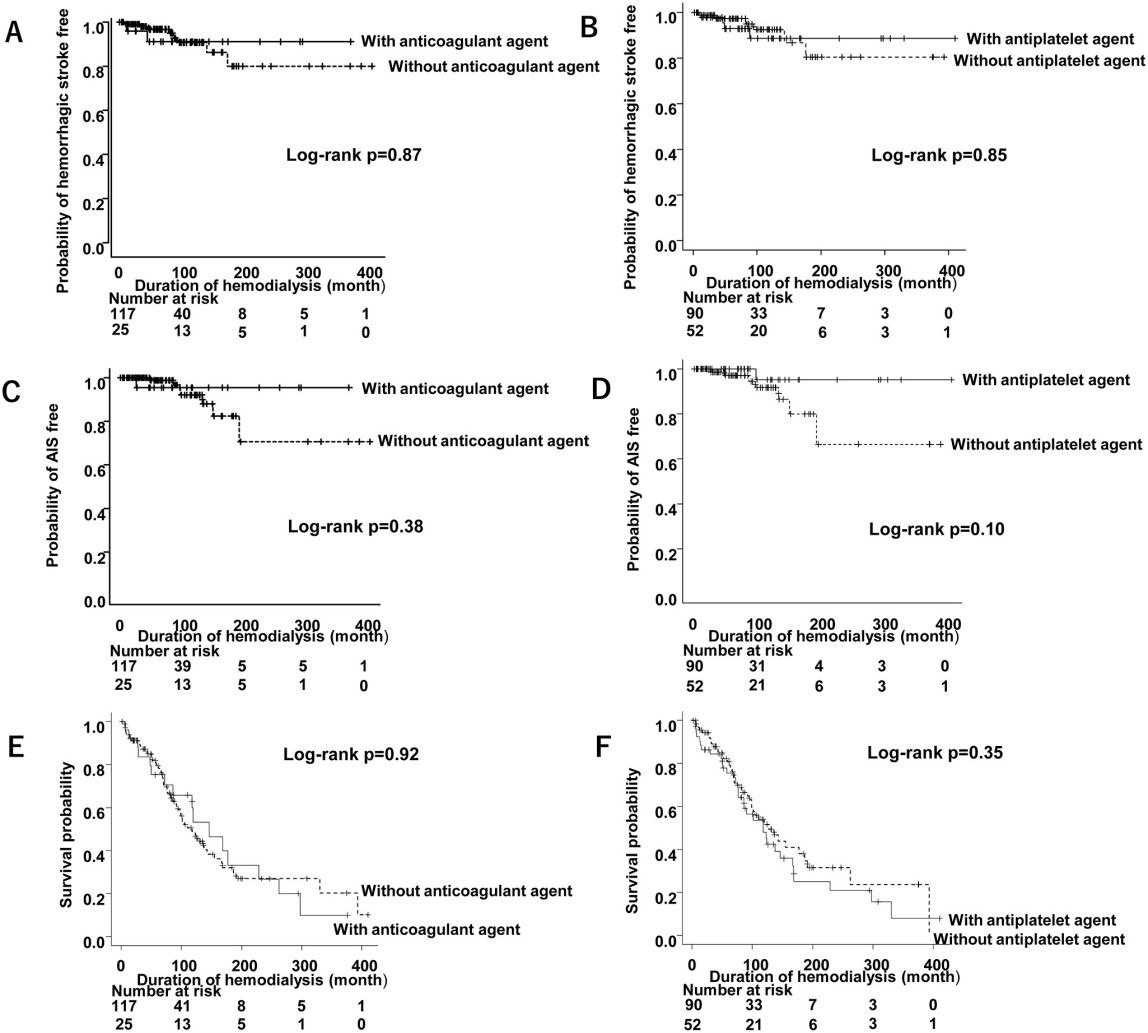
Kaplan–Meier curves of the (A, B) hemorrhagic stroke-free rate, (C, D) AIS-free rate, and (E, F) survival probability with or without anticoagulant/antiplatelet use. There were no significant differences with or without anticoagulant/antiplatelet use. AIS, acute ischemic stroke.

We also examined the Kaplan–Meier curves of the hemorrhagic stroke-free rate (Fig 4A and 4B), AIS-free rate (Fig 4C and 4D), and survival probability (Fig 4E and 4F) with or without anticoagulant/antiplatelet use; however, there were no significant differences.

**Fig 5:**
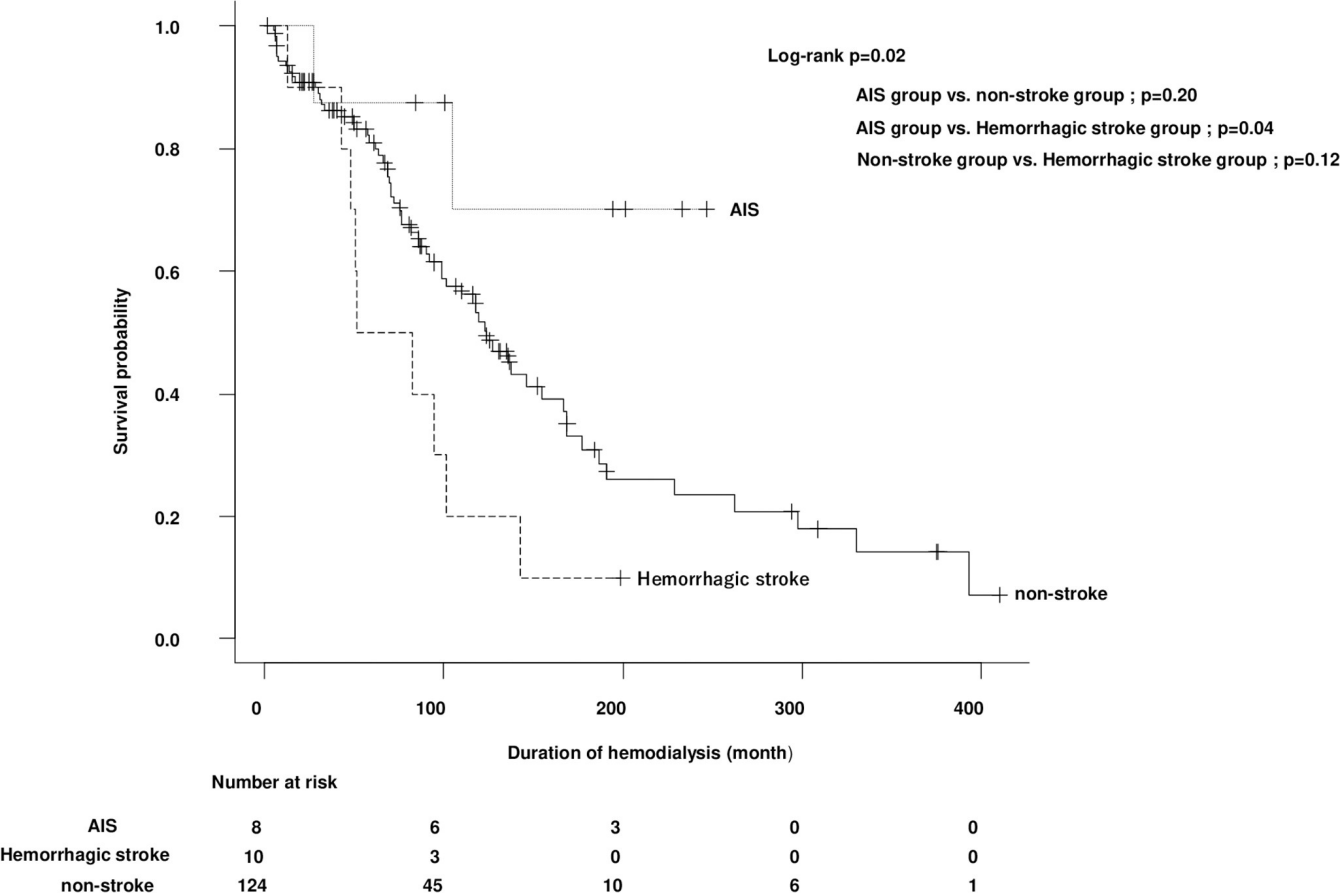
Survival probability of hemodialysis patients. The AIS group was not associated with lower survival probability than the non-stroke group or the hemorrhagic stroke group, but the hemorrhagic stroke group had significantly lower survival probability than the AIS group (p = 0.04). AIS, acute ischemic stroke.

The AIS group was not associated with a lower survival probability than the non-stroke group or the hemorrhagic stroke group. However, the hemorrhagic stroke group had significantly lower survival probability than the AIS group (p = 0.04) (Fig 5).

## Discussion

Hospitals on remote islands in Nagasaki, Japan, including our hospital, have an effective drip-and-ship (DS) system. A teleradiology system and helicopter transport system, established in the region to transport emergency patients with AIS from these islands to hub centers at any time, has demonstrated effectiveness, including in the use of mechanical thrombectomy devices [15,16].

According to the Japanese Guidelines for the Management of Stroke 2021 [17], two hemorrhagic stroke patients in this study were indicated for surgery. Similarly, three of the eight patients with AIS were eligible for rt-PA administration, and one was eligible for endovascular treatment. However, none of the patients were transported by helicopter, and none received these treatments, because they were undergoing hemodialysis while living on remote islands.

In hemodialysis patients, the hematoma is larger, and the mortality rate is twice as high as in those with normal renal function [18]. The most common site of cerebral hemorrhage is the basal ganglia, similar to that in non-hemodialysis patients [19]. The putamen was also the most common site of hematoma in our study, and the size was quite large. Most patients were difficult to transport, because they had extremely severe conditions at the time of onset; thus, the mortality rate and the number of cases with severe disability (mRS 5/6) were extremely high.

Currently, in the remote island region of Nagasaki, all patients requiring rt-PA administration are to be transported by helicopter to a hub center on the mainland because of the lack of facilities for neurosurgery on remote islands. Apart from considering the indications for rt-PA in patients with severe renal impairment, hemodialysis patients living on remote islands must be considered at risk of sudden changes in status during transportation. Thus, rt-PA use was postponed in some cases, leading to none being transported by helicopter. However, in AIS patients, although the mortality rate was not as high as that in hemorrhagic stroke patients, the proportion of patients with moderate disability (mRS 2–4) was increased. Thus, rt-PA may be used aggressively despite these risks.

There are no stroke specialists or facilities on many remote islands, and acute stroke treatment is subject to distance and time constraints. Thus, similar to establishing transport systems, such as the drip-and-ship system, prevention and risk management of stroke is important, especially for hemodialysis patients who are at high risk of stroke.

In addition to vascular complications in hemodialysis patients, other complications include blood pressure abnormalities, renal osteodystrophy, renal anemia, infections, arteriosclerosis, and atrial fibrillation [20,21]. To prevent these complications, prophylactic treatment using anticoagulant and antiplatelet agents may sometimes be needed. Our study showed that hemorrhagic stroke has a poor neurological outcome and high mortality. Thus, its prevention is critical.

Although we used warfarin, there are few reports on the effectiveness of warfarin in hemodialysis patients, and there is no consensus in this regard. Some studies report that the risk of developing AIS is reduced with warfarin administration, while others report an increased risk of ICH [22,23].

We observed no significant differences in the probability of hemorrhagic stroke, AIS, and survival probability with anticoagulant/antiplatelet use. Our results showed that once hemorrhagic stroke developed, the hematoma could be enormous, causing death and decreased survival probability. In addition, two of the hemorrhagic stroke patients who were taking both anticoagulant and antiplatelet agents died. Thus, anticoagulant use, which is particularly controversial and failed to show a protective effect against AIS in this study, may require careful consideration.

Excess body fluid and activation of the renin–angiotensin–aldosterone system and sympathetic nervous system decreased blood levels of antihypertensive drugs due to hemodialysis. Dysfunction of vascular endothelial cells has been reported as a cause of hemorrhagic stroke in hemodialysis patients [24,25]. Moreover, in our study, 90% of patients with hemorrhagic stroke had hypertension, suggesting that blood pressure control is crucial for the prevention of hemorrhagic stroke in hemodialysis patients.

Agarwal et al. [26] reported that the increase in blood pressure during hemodialysis was improved by setting an appropriate dry weight. In addition to preventing lifestyle-related diseases, management during hemodialysis is also essential. Similar to stroke care, maintenance hemodialysis is also specialized medical care that requires supervision by a specialist. However, hemodialysis facilities that need patients to visit the hospital three times a week cannot focus only on the mainland and must respond locally too. Therefore, the participation of generalists in specialized medical care in the community, the support of specialists for this, and appropriate dialysis management through frequent cooperation with paramedics is important for preventing stroke, especially hemorrhagic stroke.

Our study has some limitations, including its retrospective design and small study population. As this was a retrospective cohort study in only one remote island area, more extensive prospective studies may be required in the future.

## Conclusions

This is the first study to report the status of stroke in hemodialysis patients living on remote islands, thus providing valuable information for improved stroke management in such patients.

## Supporting information

S1 Checklist STROBE statement—Checklist of items that should be included in reports of observational studies. (DOCX)
